# *Lumbriculus variegatus*: A novel organism for in vivo pharmacology education

**DOI:** 10.1002/prp2.853

**Published:** 2021-08-20

**Authors:** Aidan Seeley, Caitlin Bellamy, Nia A. Davies, Melisa J. Wallace

**Affiliations:** ^1^ Swansea Worm Integrative Research Laboratory (SWIRL) Swansea University Medical School Swansea University Swansea United Kingdom

**Keywords:** animals, laboratory, education, invertebrates, models, animal, models, educational, oligochaeta, teaching

## Abstract

Pharmacology graduates require an understanding of both in vitro and in vivo drug responses but there has been a decline in animal use in pharmacology education over the last 30 years. To address this, we present the novel invertebrate model, *Lumbriculus variegatus*, for in vivo testing of drugs in a teaching environment. We have developed two novel behavioral assays: the stereotypical movement assay, which measures the effect of drugs on the ability of *L*. *variegatus* to perform stereotypical movements following tactile stimulation, and the free locomotion assay, which measures drug effects on unstimulated movement. We report the effects of compounds with diverse pharmacodynamic properties on *L*. *variegatus* using these assays. The ryanodine receptor antagonist, dantrolene, altered the unstimulated movement of *L*. *variegatus* at 5 μM, whereas stimulated movement was inhibited at ≥25 μM. Lidocaine, a voltage‐gated sodium channel blocker, and quinine, a nonselective sodium and potassium channel blocker, reduced both stimulated and unstimulated *L*. *variegatus* movement at ≥0.5 mM. Inhibitory effects of quinine persisted for up to 24 h after drug removal, whereas lidocaine effects were reduced 10 min after drug removal. Herein, we provide proof‐of‐concept utilization of *L*. *variegatus* as an organism for use in in vivo pharmacology education but without regulatory constraints or the need for specialized equipment and training.

## Graphical abstract

*Lumbriculus variegatus* were used as a novel organism for studying the effects of drug compounds on stereotypical behaviors and unstimulated movement for use within in vivo pharmacology education.

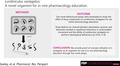

## 1 INTRODUCTION

Pharmacology is the study of how medicines and other drugs work and are processed by the body.^1^ As such, pharmacology undergraduates require a comprehensive understanding of both in vitro and in vivo drug responses. Despite advances in molecular biologic techniques, studies in isolated cells and tissues do not fully model the complex interactions observed in whole organisms. Currently, in vivo validation remains critical in the drug discovery and development process.^2^ In order to limit the use of animal models at this preclinic stage, the principles of replacement, reduction, and refinement, the 3Rs,^3^ aims to address the potential harms to animals while supporting high‐quality science and translation by addressing the benefits of this research.^4^

Animal use in pharmacology education has steadily declined in the last 30 years, with their use in education and training accounting for <1% of in vivo experimental procedures in 2019^5^ and <2% of students taught in vivo skills during their degree.^6^

Learned societies, such as the British Pharmacologic Society, have highlighted the importance of these skills through inclusion in recommended curricula within pharmacology education.^7^, ^8^ Despite significant financial contributions from these societies and the pharmaceutical industry, in vivo skills remain an area of concern.^9^, ^10^, ^11^, ^12^

We present a novel invertebrate model, *Lumbriculus variegatus*, for use in whole‐organism studies in a teaching environment. Excluding any living cephalopod, invertebrates are not covered under the Animal (Scientific Procedures) Act 1986, and this organism, therefore, offers the opportunity for utilization within pharmacology education.

*L*. *variegatus* is an aquatic worm inhabiting shallow freshwater ponds, lakes, and marshes,^13^ and these animals have been extensively characterized as indicator organisms for toxic compounds in aquatic systems.^14^, ^15^, ^16^, ^17^, ^18^, ^19^ Touching the anterior of *L*. *variegatus* results in retraction and the reversal of body position, whereas touching the tail elicits helical swimming.^13^ These behaviors have previously been described and used to determine the effects of exogenous compounds on *L*. *variegatus*.^13^, ^14^, ^20^ Despite the previous works of literatures on ecological toxicology, much less is known about *L*. *variegatus* reaction to drug compounds.^21^, ^22^

*L*. *variegatus* enables the inclusion of practical in vivo pharmacology experiments to improve student learning and confidence within the laboratory as well as training in in vivo pharmacology. This organism is low‐cost and exempt from much of the regulation and ethical challenges associated with conventional in vivo models, which often prevent in vivo practical classes.^6^, ^23^

Using novel assays which have been developed to be easily transferred for inclusion within the education setting, we present the effects of three distinct ion channel blockers on the behavior of *L*. *variegatus*. Specifically, the ability to perform the stereotypical behaviors of body reversal and helical swimming following tactile stimulation and unstimulated free locomotion, in the presence of dantrolene, a ryanodine receptor antagonist,^24^ lidocaine, a voltage‐gated sodium channel blocker,^25^ and quinine, a nonselective sodium and potassium channel blocker.^26^ Our aim was to develop a novel whole animal model for use within a teaching environment for the demonstration of fundamental pharmacologic principles and techniques. This was tested in a first‐year medical pharmacology laboratory practical, and anecdotal feedback collected, alongside experimental data.

We found that *L*. *variegatus* is a technically straightforward yet effective animal model for the teaching of in vivo pharmacology. Additionally, this organism has broader potential in pharmacologic education, analogous to *Caenorhabditis elegans* and *Drosophila melanogaster*.

## 2 METHODS

### 2.1 *Lumbriculus variegatus* culture

*L*. *variegatus* were purchased from Alfa Fish Foods and laboratory‐reared in aquariums containing artificial pondwater, composed as previously described by O’Gara et al.,^14^ using UV‐treated deionized water produced by Elix® Essential 3 UV Water Purification System. The artificial pondwater was continuously aerated and water filtered using commercial air stones and aquarium filters, respectively. The pH was not monitored or adjusted once the worms were placed in the water. The aquariums were kept at room temperature (18–21°C) and subject to a 16:8‐h light‐dark cycle. Cultures were fed TetraMin flakes and 10 mg/L spirulina weekly.

*L*. *variegatus* populations were maintained for a minimum of 3 months before experimentation to limit variation in the colony. Individual worms used in experiments were randomly selected, lacked any obvious morphological defects, and ranged from 2 to 8 cm in length as per previous studies^14^ as we observed no size‐dependent changes within this range.

### 2.2 Reagents and solutions

Dantrolene, lidocaine, and quinine were obtained from Sigma‐Aldrich (Dorset, United Kingdom). Dantrolene and quinine were dissolved in 100% dimethyl sulfoxide (DMSO) (Sigma‐Aldrich) for a stock solution concentration of 10 mM and 200 mM, respectively. Dantrolene and quinine were diluted in artificial pondwater to give a final DMSO concentration of 0.5% and a maximum final concentration of 50 μM and 1 mM, respectively. Artificial pondwater with 0.5% DMSO was used as a vehicle control for dantrolene and quinine experiments. Lidocaine was dissolved in artificial pondwater to give a maximum of 1 mM final concentration and artificial pondwater was used as the vehicle control.

### 2.3 Stereotypical movement assay

**FIGURE 1:**
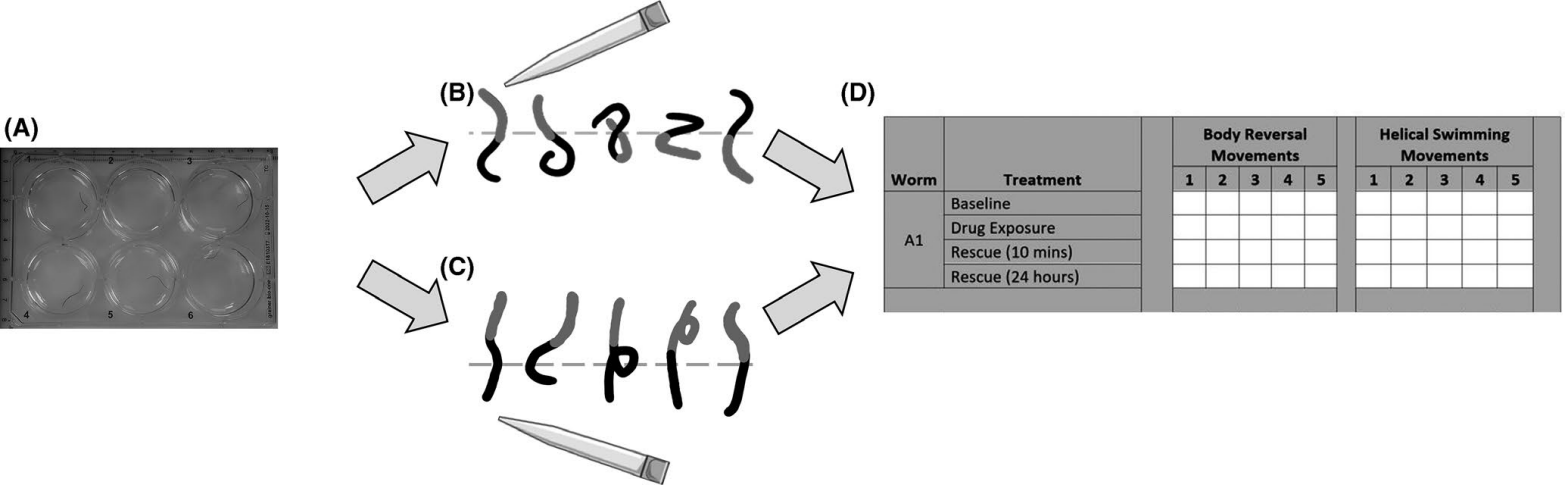
Measuring stereotypical movement of *Lumbriculus variegatus*. (A) *L*. *variegatus* are plated in 6‐well plates 18–24 h before the experiment begins. *L*. *variegatus* are alternately stimulated with 20–200 μl pipette tip at the (B) anterior region (shown in gray), to stimulate body reversal, or (C) posterior region (shown in black), to stimulate helical swimming. Worms are stimulated for a total of 5 times/end, with a 5–10‐s interval between stimuli. (D) These movements are objectively scored and recorded as 1 = no movement, 2 = incomplete stereotypical movement, 3 = full stereotypical movement, as previously described by Drewes.^13^ (A–D) is repeated for each *L*. *variegatus* before exposure to drug compounds to give the baseline ability to perform these movements. *L*. *variegatus* are then tested again 10 min after incubation with drugs and 10 min and 24 h in artificial pondwater only. Data are expressed as a ratio of the movement score after exposure relative to the baseline movement score

Eighteen to 24 h before experimentation, one *L*. *variegatus* worm was placed in each well of a Cellstar® 6‐well plate (Greiner Bio‐One) containing 4 ml of artificial pondwater. Plates were kept at room temperature and subject to a 16:8‐h light‐dark. After this acclimation period, the pondwater was replaced and the baseline ability of the worm to perform stereotypical behaviors was tested and recorded (Baseline). This was done by alternately stimulating the anterior or posterior ends of the body with a clean 20–200 μl plastic pipette tip, 5 times per end, with a 5–10‐s interval between stimuli. Movement was scored as 1 = no movement, 2 = incomplete stereotypical movement, 3 = full stereotypical movement. The artificial pondwater was then removed and immediately replaced with a drug or a vehicle (artificial pondwater only or 0.5% DMSO in artificial pondwater) was added. After a 10‐min incubation with a drug solution or vehicle, the worms were tested again using the same procedure (drug exposure). For the “rescue” experiments, the drug solution or vehicle was aspirated from the well, and to remove any latent drug or vehicle residue, fresh pondwater was added and then immediately aspirated and then replaced with fresh, untreated pondwater. These worms were then retested at 10 min (Rescue 10 mins) and 24 h (Rescue 24 h) postdrug or vehicle treatment. Data are expressed as a ratio of the movement score while in treatment relative to baseline. The data collection methods for these assays are shown in Figure 1.

### 2.4 Free locomotion assays

As in the stereotypical movement assay, 18–24 h before testing, worms were placed individually in Cellstar® 6‐well plates with fresh pondwater and kept at room temperature and subject to a 16:8‐h light‐dark. Following this acclimation period, pondwater was replaced with 2 ml fresh artificial pondwater to limit movement in the *z*‐axis, and baseline free locomotion was recorded by rapid, sequential image collection with a 13‐megapixel camera at a rate of one image per second for 50 s. Images were then collected 10 min after removing and immediately replacing artificial pondwater wiperth a drug solution or vehicle. Drug solutions and vehicle controls were then removed, the wells washed, and fresh pondwater added. Rescue experiments were collected at 10 min (Rescue 10 mins) and 24 h (Rescue 24 h) after drug or vehicle removal.

**FIGURE 2:**
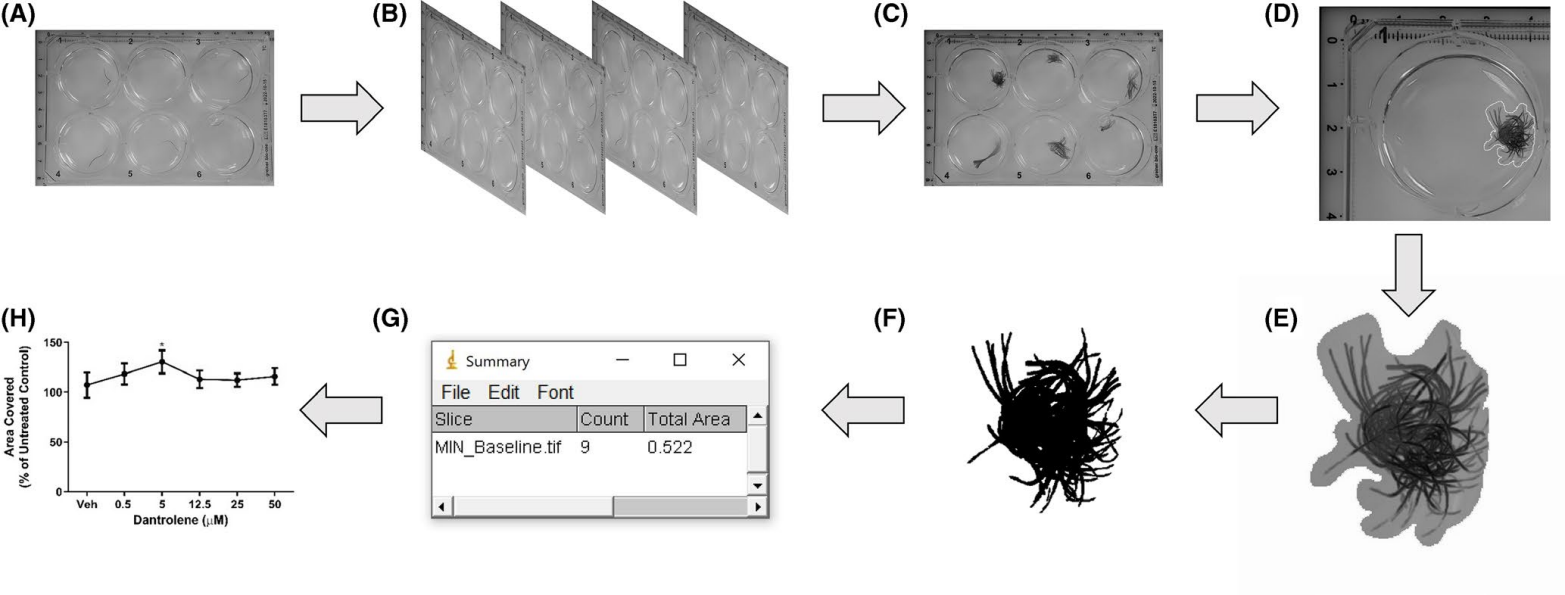
Measuring free locomotion of *Lumbriculus variegatus*. (A) *L*. *variegatus* are plated in 6‐well plates 18–24 h before the experiment begins. (B) 50 images are then collected at 1‐s intervals. (C) Images are then arranged into a *z*‐stack and scale is set to an area of known distance within the z‐stack. (D) Each individual *L*. *variegatus* is isolated using freehand selection and (E) isolated from the remaining image. (F) Thresholds are then set to only select *L*. *variegatus* and background is then removed. (G) The total area covered by each *L*. *variegatus* can then be calculated using the set scale and (H) graphed for presentation and analysis. (A–F) is repeated for each *L*. *variegatus* to give the baseline movement before exposure to drug compounds, 10 min after incubation with drugs and 10 min and 24 h in artificial pondwater only. Data are expressed as a percentage of baseline controls

Collected images were analyzed using ImageJ software. These images were compiled into a *z*‐stack image, this being a compilation of photographs taken at 1‐s intervals over 50 s. An area of known distance within each z‐stack image was measured and ImageJ calibrated to pixels per centimeter (pixels/cm) within each image. To determine the area traversed by each worm, the foreground and background were separated using the thresholding functionality of ImageJ to separate the pixels activated by *L*. *variegatus* from those activated by the 6‐well plate. The total area covered by the *L*. *variegatus* at baseline, drug exposure, Rescue 10 min, and Rescue 24 h was then determined based on the calibration of pixels/cm within ImageJ. Data are expressed as a percentage of the area covered by *L*. *variegatus* in baseline conditions. The data collection method for this assay is shown in Figure 2.

For both assays and as per previous studies that have used *L*. *variegatus*,^14^ decompositions, as determined by visible tissue degeneration and whole‐organism tissue pallor, at assay endpoints was the main indicator of lethal toxicity. *L*. *variegatus* were only exposed to one test compound and euthanized at assay endpoints by rapid submersion in 70% ethanol.

### 2.5 Statistical analysis

The sample size for each assay and treatment was eight worms. Data are displayed as the mean ± standard error of the mean (SEM) for each data set. Data are relative to the untreated, baseline control condition. Values for each behavioral measurement were compared with the untreated control conditions (baseline) for each *L*. *variegatus* per condition. Statistical analysis was performed in GraphPad Prism 9. Drug exposure conditions were compared with baseline conditions by paired nonparametric two‐tailed *t* test for stereotypical movement assays and paired parametric two‐tailed *t* test for free locomotion assays. A two‐way ANOVA with Dunnett's posttest was used to analyze 10‐min and 24‐h rescue time points compared with baseline conditions for both assays. *p* < .05 was the threshold for statistical significance.

## 3 RESULTS

### 3.1 Behavioral response to dantrolene

**FIGURE 3:**
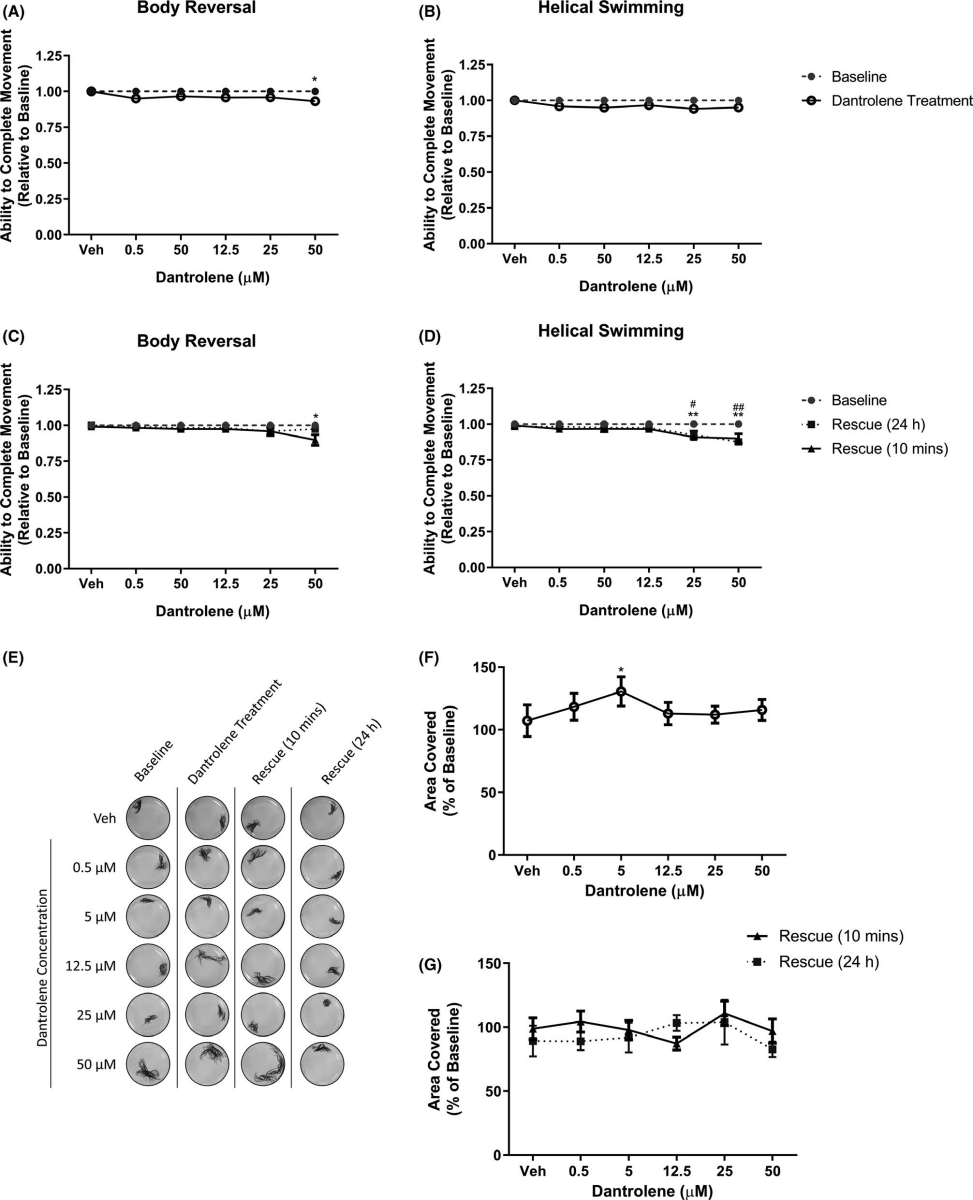
The effect of dantrolene on *Lumbriculus variegatus* behavior. *L*. *variegatus* were exposed to dantrolene (0–50 µM) and tested for the ability of tactile stimulation to elicit (A) body reversal or (B) helical swimming. Dantrolene was then removed and the ability of *L*. *variegatus* to perform (C) body reversal or (D) helical swimming was tested after 10 min and 24 h. Data are expressed as a ratio of the movement score after exposure relative to the movement score at baseline. (E) The effect of dantrolene on free locomotion was measured before dantrolene exposure (Baseline), after 10 min of exposure to 0–50 µM dantrolene (Dantrolene Treatment), 10 min after dantrolene removal (Rescue 10 min), and 24 h after dantrolene removal (Rescue 24 h). Quantification of the area covered by *L*. *variegatus* following (F) dantrolene treatment and (G) removal of dantrolene for 10 min and 24 h are expressed as a percentage of the area covered at baseline. Error bars represent the standard error of the mean, *n *= 8 for each concentration. Veh: 0.5% DMSO in artificial pondwater. */# *p* < .05, **/## *p* < .01; where * refers to statistical significance between baseline and dantrolene exposure or statistical significance between baseline and rescue 10 mins, # refers to statistical significance between baseline and rescue 24 h

The ryanodine receptor antagonist, dantrolene,^24^ had no significant effects on stereotypical movements at ≤25 µM. However, we found that 50 µM minimally but significantly inhibited body reversal after 10 min exposure (*p *= .0313, Figure 3A). This was not the case for helical swimming (*p* > .05, Figure 3B). Ten min after removal of 50 µM dantrolene and incubation in artificial pondwater, body reversal was significantly reduced compared with baseline (*p *= .0121, Figure 3C). We also observed that helical swimming movements were reduced 10 min after the removal of 25 µM (*p *= .0088, Figure 3D) and 50 µM dantrolene (*p* = .0081, Figure 3D). These effects persisted for 24‐h after exposure to both 25 µM (*p *= .0290, Figure 3D) and 50 µM dantrolene (*p *= .0015, Figure 3D) but were only observed for the helical swimming and not body reversal. Despite this prolonged effect on movement, at 24 h, the worms were still alive with no signs of tissue decomposition.

The helical swimming and body reversal assays rely on tactile stimulation by an observer. In addition to stimulated behaviors, we also wanted to determine if unstimulated free locomotion was affected by our test compounds. Figure 3E–G shows that for dantrolene, we observed no significant differences between baseline free locomotion and drug treatment (Figure 3F and G) except for 5 µM. This treatment significantly increased free locomotion by 18.30 ± 10.85% compared with baseline (*p *= .0341, Figure 3E and F).

### 3.2 Behavioral response to lidocaine

**FIGURE 4:**
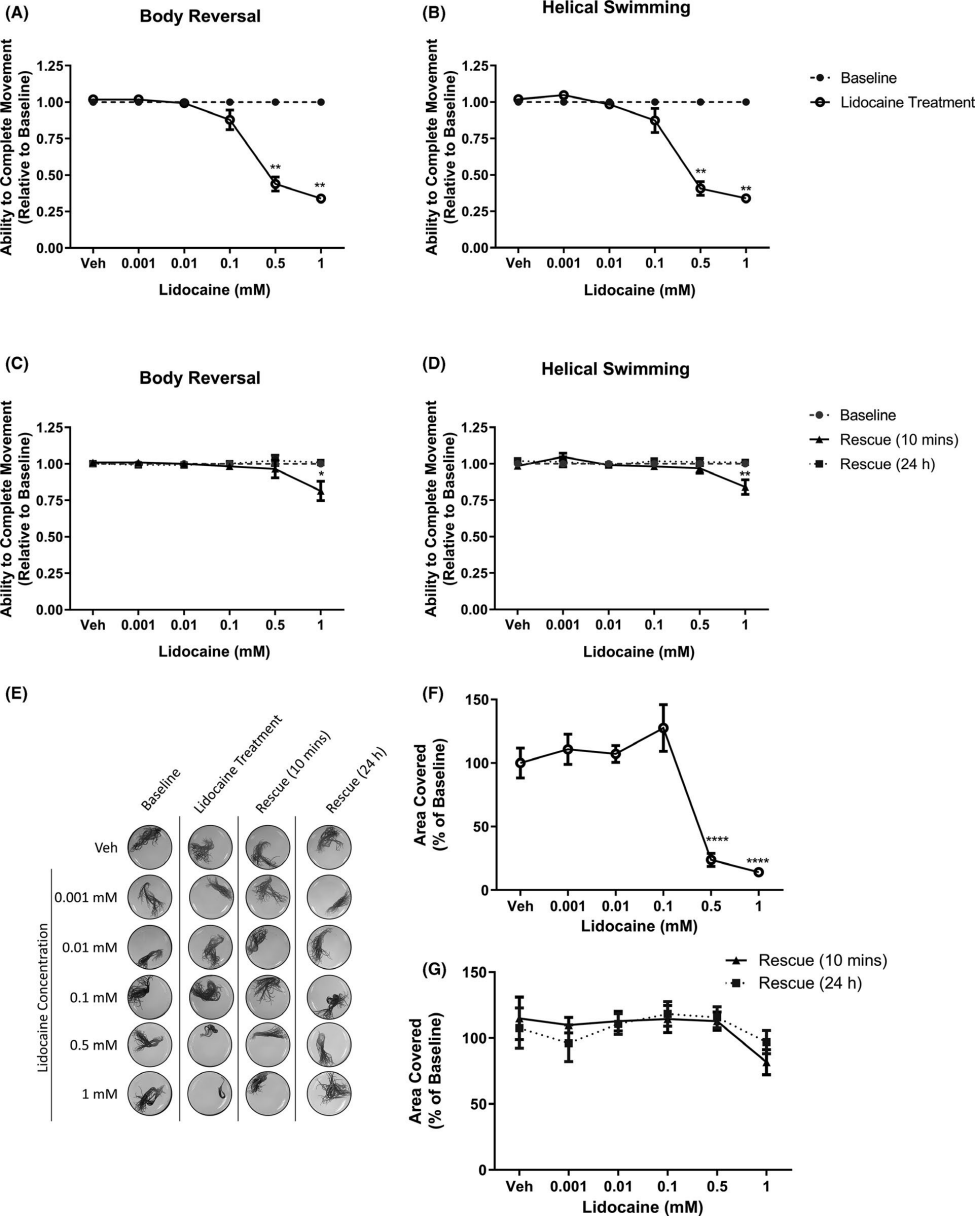
The effect of lidocaine on *Lumbriculus variegatus* behavior. *L*. *variegatus* were exposed to lidocaine (0–1 mM) and tested for the ability of tactile stimulation to elicit (A) body reversal or (B) helical swimming. Lidocaine was then removed and the ability of *L*. *variegatus* to perform (C) body reversal or (D) helical swimming was tested after 10 min and 24 h. Data are expressed as a ratio of the movement score after exposure relative to the movement score at baseline. (E) The effect of lidocaine on free locomotion was measured before lidocaine exposure (Baseline), after 10 min exposure to 0–1 mM lidocaine (Lidocaine Treatment), 10 min after lidocaine removal (Rescue 10 min), and 24 h after lidocaine removal (Rescue 24 h). Quantification of the area covered by *L*. *variegatus* following (F) lidocaine treatment and (G) removal of lidocaine for 10 min and 24 h are expressed as a percentage of the area covered at baseline. Error bars represent the standard error of the mean, *n *= 8 for each concentration. Veh: Artificial pondwater. *p < .05, **p < .01, ***p < .001, ****p < .0001

As shown in Figure 4A and B, we found that the sodium channel blocker lidocaine significantly inhibited both body reversal and helical swimming at 0.5 mM and 1 mM. At concentrations ≤0.5 mM, these effects were reversed following 10 min in drug‐free artificial pondwater, with no significant difference compared with baseline (*p* > .05, Figure 4C and D). However, the effect of 1 mM lidocaine persisted 10 min after removal, significantly inhibiting both body reversal (*p* = .0115, Figure 4C) and helical swimming (*p* = .0035, Figure 4D). Twenty‐four hours after lidocaine exposure, both movements returned to baseline levels (*p* > .05, Figure 4C‐D).

We also observed these dose‐dependent effects in the free locomotion assays (Figure 4E‐G), where the movement was significantly reduced at 0.5 mM (76.24 ± 5.23%) and 1mM (85.92 ± 5.23%) lidocaine compared with baseline levels (*p* < .0001, Figure 4F). Similar to the stereotypical movement assay, movement returned to baseline levels at both 10 min and 24 h after drug exposure(*p* > .05, Figure 4G).

### 3.3 Behavioral response to quinine

**FIGURE 5:**
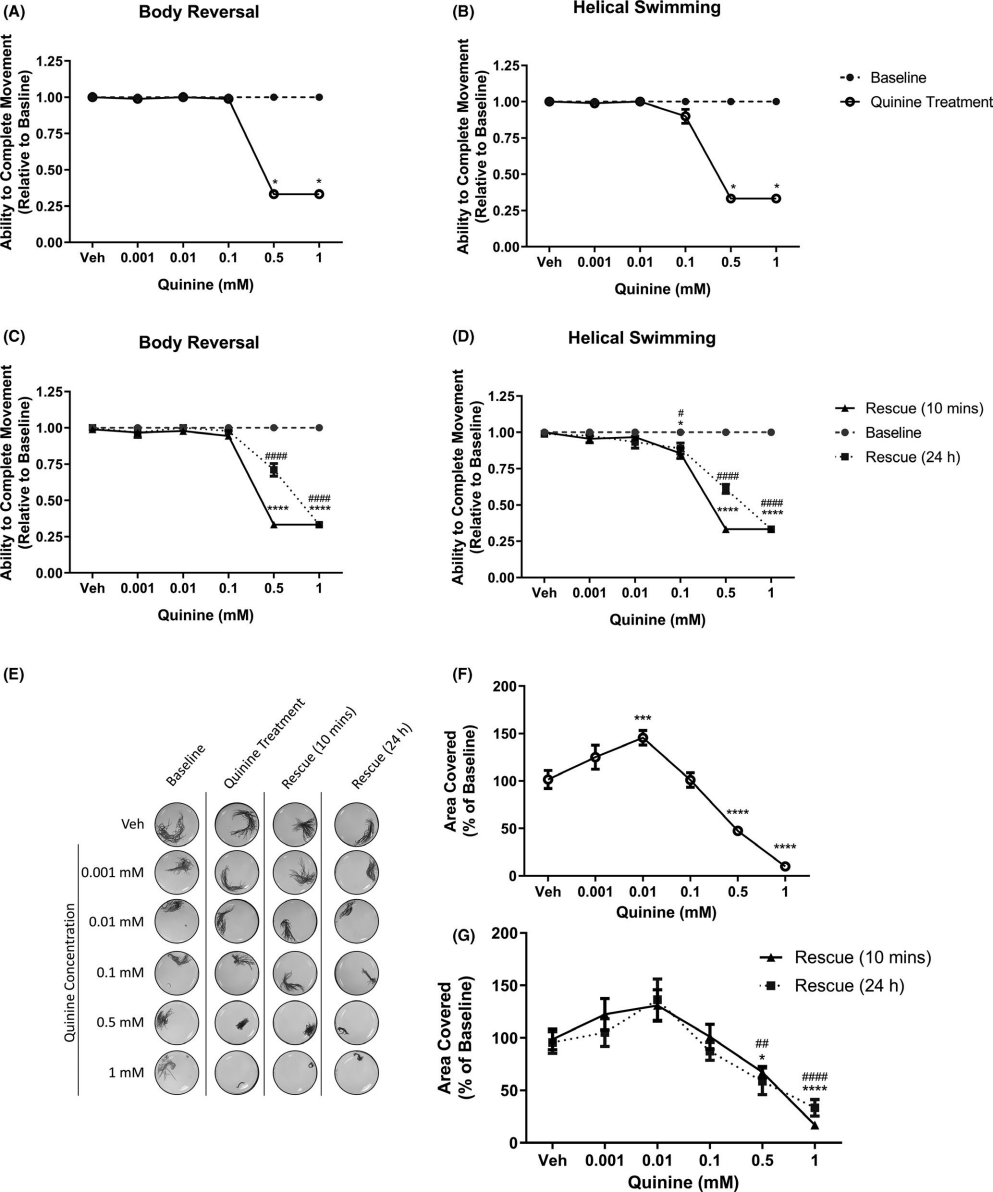
The effect of quinine on *Lumbriculus variegatus* behavior. *L*. *variegatus* were exposed to quinine (0–1 mM) and tested for the ability of tactile stimulation to elicit (A) body reversal or (B) helical swimming. Quinine was then removed and the ability of *L*. *variegatus* to perform (C) body reversal or (D) helical swimming was tested after 10 min and 24 h. Data are expressed as a ratio of the movement score after exposure relative to the movement score at baseline. (E) The effect of quinine on free locomotion was measured before quinine exposure (baseline), 10 min after exposure to 0–1 mM quinine (quinine treatment), 10 min after quinine removal (Rescue 10 min) and 24 h after quinine removal (Rescue 24 h). Quantification of the area covered by *L*. *variegatus* following (F) quinine treatment and (G) removal of quinine for 10 min and 24 h are expressed as a percentage of the area covered at baseline. Error bars represent the standard error of the mean, *n* = 8 for each concentration. Veh: 0.5% DMSO in artificial pondwater. */#*p* < .05, **/##*p* < .01, ***/###*p* < .001, ****/####*p* < .0001; where * refers to statistical significance between baseline and quinine exposure or statistical significance between baseline and rescue (10 min), # refers to statistical significance between baseline and rescue 24 h

Following on from lidocaine, we sought to examine the effect of a different ion channel blocker on *L*. *variegatus*. Figure 5A‐B shows that the nonspecific sodium and potassium channel blocker quinine inhibited both body reversal and helical swimming at equimolar concentrations to lidocaine (0.5 mM and 1 mM). However, unlike lidocaine, these effects persisted after 10 min and 24 h in drug‐free artificial (*p* < .0001, Figure 5C‐D).

We observed similar results in the free locomotion assay (Figure 5E‐G). Movement increased by 45.5±7.70% after exposure to 0.01 mM quinine (*p* = .0006, Figure 5F), however, this effect was reversed and returned to baseline conditions after 10 min and 24 h in drug‐free artificial pondwater (*p* > .05, Figure 5G). Conversely, free locomotion was inhibited by 52.59 ± 4.04% at 0.5 mM (*p* < .0001, Figure 5F) and 90.15 ± 1.67% at 1 mM concentrations (*p* < .0001, Figure 5F). This inhibitory effect persisted after 10 min in drug‐free artificial pondwater with 0.5 mM inhibiting movement by 32.68 ± 5.51% (*p* = .0112, Figure 5G) and by 83.04 ± 2.98% at 1 mM (*p* < .0001, Figure 5G) as well as after 24 h (*p* < .0001, Figure 5G). At this point, as with dantrolene (Figure 3), despite the prolonged effect on movement, the worms were alive with no signs of tissue decomposition.

## 4 DISCUSSION

*L*. *variegatus* has been extensively characterized as indicator organisms for toxic compounds in aquatic systems and proposed as a standard organism for sediment bioaccumulation tests.^14^, ^15^, ^16^, ^17^, ^18^, ^19^, ^27^ Herein, we demonstrated that *L*. *variegatus* has application as an effective model for the teaching of the effects of pharmacological agents on an intact system. Using two novel assays, we describe three different behavioral endpoints, body reversal, helical swimming, and free locomotion, that can be measured without the need for costly or specialized equipment, regulatory approval, or highly specialized animal housing facilities—requirements that often prevent the teaching of in vivo practical skills.^6^, ^23^ We recognize that experiments conducted in invertebrates do not replicate the complexity of higher animals and experiments conducted in invertebrates do not wholly replace studies in vertebrate species, such as mice and rats. However, grounding students in experience with *L*. *variegatus* will provide many of them with the whole animal experience they otherwise would not have while providing an excellent foundation experience for those who will go on to research higher organisms. Experience with this organism exposes students to concepts around replacement, refinement, and reduction^3^ in animal experimentation but also gives them the direct experimental experience of putting these elements into practice.

There are other invertebrate models available for use in pharmacology and biomedical sciences teaching: *C*.*elegans*, *D*. *melanogaster*, and others.^28^ However, in the classroom laboratory, *L*. *variegatus* presents some advantages over these organisms. First, the larger size of *L*. *variegatus* (50–80 mm) compared with *C*. *elegans* (~1 mm) makes it easier to view as an individual.^29^, ^30^ Second, for the assays presented, the drug exposure time and rescue time points have been demonstrated to be sufficient for several compounds tested to elicit effects and enable educators to complete these experiments within a standard laboratory practical teaching timeframe. Moreover, when implemented within first‐year undergraduate toxicology teaching, students have reported that these assays “[were] really helpful in helping me understand our course content,” they “made you think like a scientist” and that they were “stimulating and enjoyable.”

In the stereotypical movement assay (Figure 1), students can measure the effects of drugs on reducing two different behaviors (body reversal and helical swimming) without the need for a microscope or a specialist equipment, unlike other models used in teaching such as *C*. *elegans*.^31^ This assay allows students to distinguish *L*. *variegatus* that do not perform body reversal or helical swimming movements from worms that do—giving them hands‐on semi‐quantitative in vivo pharmacology training. The relative ease of the tactile stimulation application and the simplicity of the scoring system minimize the risk of misinterpretation of the movements and limit any variation between students conducting the assay. The free locomotion assay is a more sophisticated and quantitative experiment, which allows students to engage in movement recording and quantitative analysis.

Both assays offer educators the opportunity to use these experiments to engage students with in vivo measurement and scoring, data recording and interpretation, and statistical analysis. There is also excellent potential for introducing other key curriculum concepts such as experimental blinding, molarity calculations, drug solubility, and toxicology. Further assay development may yield practical teaching protocols for behavioral assays such as tolerance and place preference, in vitro assays such as receptor binding and immunohistochemistry, and drug dose–response relationship for other physiological measurements, for example, pulse rate.^32^

No in vivo model is without limitations. One constraint in studying aquatic organisms is tested compound solubility. Our ability to investigate dantrolene was limited due to its known solubility and precipitation issues.^24^, ^33^ As such, 50 µM was the maximal concentration achieved using DMSO (0.5%) in artificial pondwater. Similarly, lidocaine and quinine were used at maximal concentrations dictated by their solubility. Additionally, it should be noted that DMSO (0.5%) as a vehicle produced no significant changes in worm behavior compared with baseline (Figures 3 and 5). Further investigation into drug compound solubility and the effects of different vehicles on these worms are needed to fully identify the limitations of this specific model.

The differential acute and long‐term effects of dantrolene, lidocaine, and quinine on *L*. *variegatus* behavior suggest that these drugs are working through distinct mechanisms. It is not known if this species expresses the sites of action for their respective mechanisms of action. Currently, this is limited is by the current lack of genomic information on *L*. *variegatus*; it is full genome has not been sequenced and protein expression studies are limited.^34^ This lack of knowledge presents both limitations and opportunities for further pharmacological investigation of these animals. For example, in our study, dantrolene did not demonstrate a straightforward dose‐dependent effect on *L*. *variegatus* stereotypical movements (Figure 3A–D) or free locomotion (Figure 3E–G). The 5 µM produced locomotor activation while higher concentrations did not. This variable response may be due to a lack, or the alteration, of the dantrolene binding site within ryanodine receptors or their homologs. It may also be differential absorption, distribution, metabolism, and excretion of pharmacological compounds that may account for the varying results observed here. Studies have demonstrated that *L*. *variegatus* express the ATP‐binding cassette transporter protein, p‐glycoprotein,^35^ but further study is required to dissect the applicability of pharmacokinetics within *L*. *variegatus* to pharmacological studies.

Lidocaine and quinine, however, demonstrated clear dose‐dependent effects on both stereotypical movements (Figures 4A–D and 5A–D,) and free locomotion (Figures 4E–G and 5E–G) at 0.5–1 mM. Interestingly, quinine demonstrated a 45.5±7.70% increase in free locomotion at 0.01 mM (*p* = .0006, Figure 5F) and then effects became inhibitory. This may be due to off‐target toxicity at concentrations >0.1 mM. Throughout our assays, we observed no evidence of decomposition of *L*. *variegatus* 24 h after drug exposure, indicating that doses used were sublethal.

While we have shown that these compounds induce differential pharmacodynamic effects with lidocaine being readily reversible and quinine having long term, but sublethal, further study is required to elucidate the full pharmacokinetic and pharmacodynamic profile of this species.

*L*. *variegatus*, and the novel assays we present, will emphasize to students the use and importance of animals in pharmacological research and drug development while giving them hands‐on experience in a living system. The purpose of our study was not to provide a full evaluation of the resource by students but anecdotal comments and feedback from them indicate that they enjoy using the worms and they recognize the practical skills they have gained in doing so. It is important to ensure that pharmacology students continue to receive training in in vivo pharmacology at a time when few students currently do.^6^ Educators must seek to address the skills gap and prepare successful graduates^9^, ^10^, ^11^, ^12^—both for our students’ benefit and for the continued advancement of the pharmacology discipline.

## DISCLOSURE

The authors declare that they have no conflict of interest.

## Data Availability

The data that support the findings of this study are available from the corresponding author upon reasonable request.
